# Estrogen Receptor Ligands: A Review (2013–2015)

**DOI:** 10.3390/scipharm84030409

**Published:** 2016-04-13

**Authors:** Shabnam Farzaneh, Afshin Zarghi

**Affiliations:** Department of Medicinal Chemistry, School of Pharmacy, Shahid Beheshti University of Medical Sciences, P.O. Box 14155-6153, Tehran 19919-5338, Iran; Shabnamfa92@gmail.com

**Keywords:** estrogens, estrogen receptor α, estrogen receptor β, ligand, patent, cancer

## Abstract

Estrogen receptors (ERs) are a group of compounds named for their importance in both menstrual and estrous reproductive cycles. They are involved in the regulation of various processes ranging from tissue growth maintenance to reproduction. Their action is mediated through ER nuclear receptors. Two subtypes of the estrogen receptor, ERα and ERβ, exist and exhibit distinct cellular and tissue distribution patterns. In humans, both receptor subtypes are expressed in many cells and tissues, and they control key physiological functions in various organ systems. Estrogens attract great attention due to their wide applications in female reproductive functions and treatment of some estrogen-dependent cancers and osteoporosis. This paper provides a general review of ER ligands published in international journals patented between 2013 and 2015. The broad physiological profile of estrogens has attracted the attention of many researchers to develop new estrogen ligands as therapeutic molecules for various clinical purposes. After the discovery of the ERβ receptor, subtype-selective ligands could be used to elicit beneficial estrogen-like activities and reduce adverse side effects, based on the different distributions and relative levels of the two ER subtypes in different estrogen target tissues. Therefore, recent literature has focused on selective estrogen ligands as highly promising agents for the treatment of some types of cancer, as well as for cardiovascular, inflammatory, and neurodegenerative diseases. Estrogen receptors are nuclear transcription factors that are involved in the regulation of many complex physiological functions in humans. Selective estrogen ligands are highly promising targets for treatment of some types of cancer, as well as for cardiovascular, inflammatory and neurodegenerative diseases. Extensive structure-activity relationship studies of ER ligands based on small molecules indicate that many different structural scaffolds may provide high-affinity compounds, provided that some basic structural requirements are present.

## 1. Introduction

### 1.1. Estrogen

Estrogens are a group of compounds named for their importance in both menstrual and estrous reproductive cycles. They are primary female sex hormones. Estrogens readily diffuse across the cell membrane. Inside the cell, they bind and activate estrogen receptors.

#### Estrogen Receptor Subtypes

Two subtypes of the estrogen receptor, ERα and ERβ, exist and exhibit distinct cellular and tissue distribution patterns. In humans, both receptor subtypes are expressed in many cells and tissues, and they control key physiological functions in various organ systems such as reproductive, skeletal, cardiovascular, and central nervous systems. ERα is mainly expressed in the mammary gland, uterus, ovary (thecal cells), bone, male reproductive organs (testes and epididymis), prostate (stroma), liver, and adipose tissue. In contrast, ERβ is predominant in the prostate (epithelium), bladder, ovary (granulosa cells), colon, adipose tissue, and immune system. However, both subtypes are markedly expressed in the cardiovascular and central nervous systems. Estradiol and hormone replacement therapies target both the ERs, but this often leads to an increased risk of breast and endometrial cancers, and thromboembolism. Also, selective estrogen receptor modulators (SERMs), structurally, are various compounds that interact with intracellular estrogen receptors in different target organs as ER agonists or antagonists [1]. Ideal SERMs would possess antagonist activity in the mammary gland and uterus, and agonist activity in other target tissues that benefit from estrogen-like actions such as the cardiovascular, skeletal, and central nervous systems [2]. Alternatively, subtype-selective ligands could be used to elicit beneficial estrogen-like activities and reduce adverse side effects, based on the different distributions and relative levels of the two ER subtypes in the different estrogen target tissues mentioned above [3].

### 1.2. Potential Clinical Applications of Estrogens

#### 1.2.1. Cancer

The role of ERs in many cancers such as breast cancer, prostate cancer, and colorectal cancer has been extensively studied. Suppression of estrogen production in the body is a treatment for breast cancer. Especially in breast cancer, activation of ERα by estrogens is considered to be responsible for enhanced proliferation, whereas this is counteracted by the presence of ERβ, which exerts an anti-proliferative effect [4].

#### 1.2.2. Neuropathies

Estrogen directly influences brain function through estrogen receptors located on neurons in multiple areas of the brain. There is considerable evidence that indicates the important role of estradiol in the central nervous system (CNS) for different kinds of diseases, including pathologies associated with depression, anxiety, and Alzheimer’s disease. At neuronal synapses, estrogen increases the concentration of neurotransmitters such as serotonin, dopamine, and norepinephrine. It affects their release, reuptake, and enzymatic inactivation [5]. A recent study reported that aged female mice responded favorably to estrogen administration and exhibited greater anti-anxiety and antidepressant behavior [6].

#### 1.2.3. Cardiovascular Disease

Estrogen has a number of effects on cardiovascular function and disease. The regular production of estrogens in pre-menopausal women places them at a lower risk for cardiovascular disease relative to men. However, this cardioprotective effect vanishes after menopause [7]. Several mechanisms are involved in this cardioprotective role such as the effects of estrogen on lipoprotein levels, vasomotor function, LDL oxidation, coagulation, and collagen and elastin synthesis [8].

#### 1.2.4. Osteoporosis

Osteoporosis is a major health problem associated with estrogen deficiency in postmenopausal women. Many studies have indicated that estrogens play an important role in bone homeostasis, not only in women but also in men [9]. Estrogen replacement therapy (ERT) is clinically used to prevent osteoporosis.

## 2. Estrogen Receptor Ligands

ERs are nuclear transcription factors that are involved in the regulation of many complex physiological functions in humans. The term SERM describes synthetic ER ligands that display tissue-selective pharmacology. This concept was first described in 1990 [10]. As anti-estrogens, they oppose the action of estrogens in certain tissues while mimicking the action of endogenous estrogens in others. Over the past few years, many reviews have covered the progress in the development of estrogen receptors [11,12,13,14,15]. We herein provide an overview and update of compounds that have been recently reported as estrogen receptor ligands, with a particular focus on their potential clinical applications.

## 3. Steroidal Ligands

**Figure 1:**
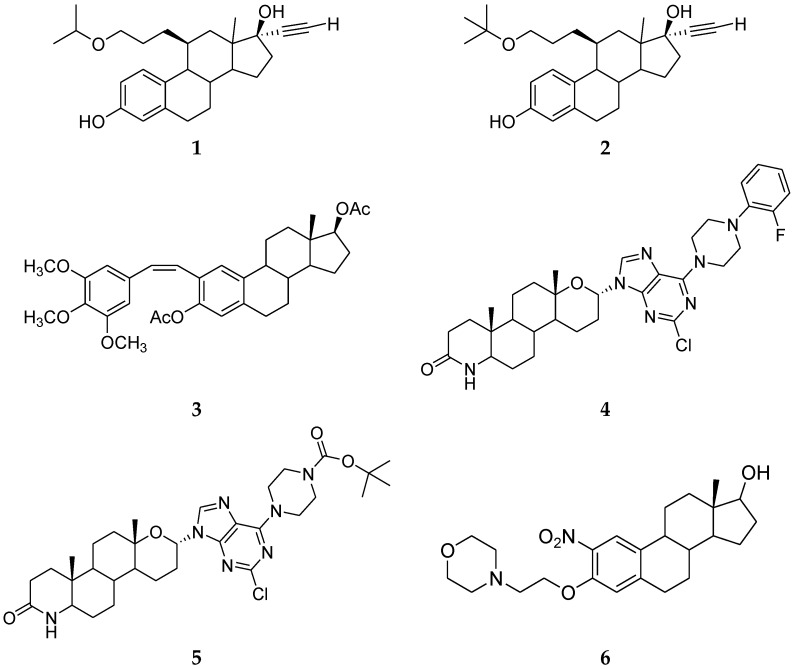
Steroidal Ligands.

Zhang et al. reported a new series of 11β-ether-17α-ethinyl-3,17ß-estradiol that demonstrates strong ER antagonist activity. The ethers were particularly interesting as they were highly active anti-estrogens that were more stable than other substituents. Among these ethers, compounds **1** and **2** (Figure 1) bind very strongly to both ERα and ERβ [16].

Combretastatin A4 analogues on a steroidal framework were reported to have anti-breast cancer properties. Recently, natural compounds have increasingly been used in traditional medicines. Combretastatin A4 is one of these compounds that induce apoptosis and act as an antiangiogenic agent [17]. Twenty-two analogues were synthesized by the Combretastatin A4 steroidal framework and among them, compound **3** (Figure 1) was the most active in both MCF-7 and MDA-MB-231 cells with IC_50_ 7.5 μM and 5.5 μM [18].

Recently, Huang et al. reported two new series of novel C6-piperazine-substituted purine steroid–nucleosides and C6-cyclo secondary amine-substituted purine steroid-nucleoside analogues. Nucleoside analogues showed an important role in the development of antitumor drugs [19,20,21]. Among them, C6-aminopurine derivatives had a wide range of biological properties and displayed antitumor activity [22,23,24]. In addition, piperazine derivatives showed a broad spectrum of biological activities, such as anticancer activity [25,26]. Therefore, these analogues were synthesized and evaluated for their anticancer activity. Among C6-piperazine-substituted purine steroid–nucleosides analogues, compound **4** (Figure 1) showed the best results on the MCF-7 cell line [27]. However, compound **5** (Figure 1) displayed only moderate anticancer activity against the MCF-7 cell line [28].

Bandyopadhyay et al. [29] also reported a new series of 2- and 4-nitroestradiol derivatives. They showed that compound **6** (Figure 1), as well as a known ER-targeting drug, 4-hydroxytamoxifen (4-HT), reduced the viability of ER-positive and ER-negative cells with similar efficacy. The toxicity exhibited by **6** was slightly better than 4-HT, with an IC_50_ value of approximately 2 μM in breast cancer cells. These data indicate that selective novel nitroestradiol compounds effectively cause cytotoxicity in breast cancer cells and their cytotoxicity might occur in an ER-independent manner.

## 4. Non-Steroidal Ligands

### 4.1. 2,3-Diaryl Isoquinolinone

**Figure 2:**
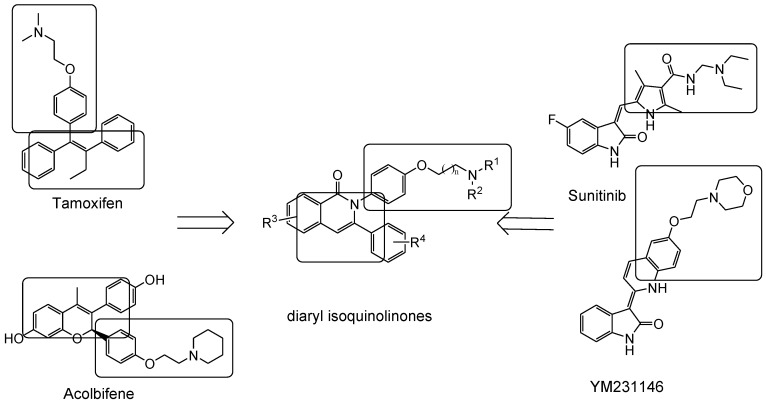
Chemical structures of SERMS, VEGFR-2 inhibitors, and designed compounds [31].

**Figure 3:**
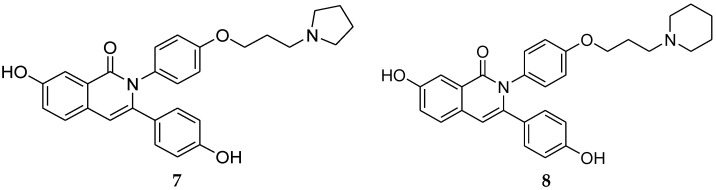
2,3-Diaryl isoquinolinone derivatives.

A new series of 2,3-diaryl isoquinolinone derivatives were reported as anti-breast cancer agents targeting ERα and vascular endothelial growth factor receptor 2 (VEGFR-2). ERα is responsible for estrogen-induced proliferation in breast cancer in which angiogenesis plays an important role in both local tumor growth and distant metastasis in many cancers [30]. Therefore, Tang et al. [31] reported a new series of compounds with characteristics of both selective estrogen receptor modulators and VEGFR-2 inhibitors. It was anticipated for these compounds with dual targets to gain more efficiency as anti-breast cancer agents with fewer side effects. VEGFR-2 inhibitors possessing an indol-2-one scaffold, such as sunitinib and YM231146, bear some structural similarities with SERMs (Figure 2). All compounds possess an aromatic scaffold and flexible side chain with a tertiary amine substituent on the end. Based upon that, 2,3-diaryl isoquinolinone derivatives were synthesized and evaluated. Among these series, compounds **7** and **8** (Figure 3) had the best affinity for ERα and the inhibition rates were as potent as tamoxifen. The results of a VEGFR-2 kinase inhibition assay showed that these derivatives had moderate-to-strong inhibitory activities compared with sunitinib. Compound **8** showed the best results for both ERα and VEGFR-2 kinase inhibition.

### 4.2. Diphenylmethane Skeleton and Related Analogues

**Figure 4:**
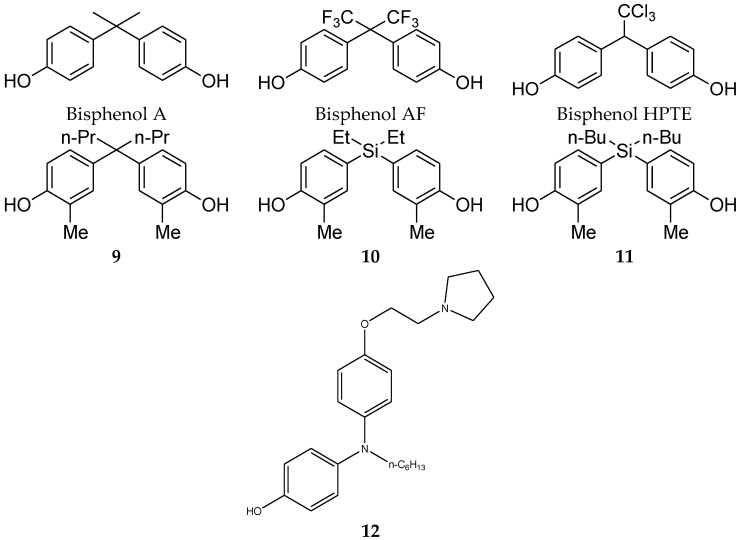
Diphenylmethane skeleton analogues.

Maruyama et al. [32,33] have shown that the diphenylmethane skeleton can have a steroid role. Bisphenol A has estrogenic activity and bisphenol AF and HPTE have agonist activity for ERα with antagonist activity for ERβ (Figure 4). Therefore, these compounds would bind to ERα in a similar manner to 17β-estradiol, but selectively for ERβ. Many analogues of bisphenol A were also synthesized and their agonist and antagonist activities were evaluated. Results showed that longer linear alkyl chains at the central carbon decreased the agonistic activities for both ERα and ERβ, but increased the antagonist activities for both ERα and ERβ. Among these series, compound **9** (Figure 4) displayed potent ERα-antagonist activity with a 28-fold selectivity over ERβ. As described, central alkyl chains were critical for potent ER antagonistic activities, indicating that the hydrophobicity at the central moiety is important for strong antagonistic activity. On the other hand, sila-substitution (C/Si exchange) of existing drugs is an attractive approach to find new drug candidates. Because silicon-containing analogues are more lipophilic and larger in molecular size than their carbon analogues [34,35,36,37], a new series of sila-analogues were synthesized with the aim of increasing hydrophobicity or molecular size. However, both sila-analogues **10** and **11** (Figure 4) showed decreased activities compared with carbon derivatives [38].

Also, in 2015 Ohta et al. [39] reported the diphenylamine skeleton as ER antagonist candidates containing a basic alkylamino side chain on one of the two phenol groups of the diphenylamine agonist core structure. The results indicated that compounds with cyclic alkylamine showed higher ER-antagonistic activity than the corresponding acyclic derivatives in a cell proliferation assay using the MCF-7 cell line. Compound **12** showed the highest antiestrogenic activity (IC_50_ = 1.3 × 10^−7^ M), being 10 times more potent than tamoxifen.

### 4.3. Deoxybenzoin Analogues

**Figure 5:**
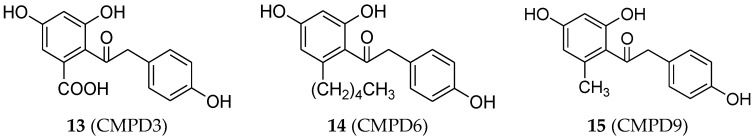
Deoxybenzoin derivatives.

Estrogenic effects of three substituted deoxybenzoins were reported by Chandrasekharan et al. [40]. These compounds were designed as CMPD3 (**13**, Figure 5), CMPD6 (**14**, Figure 5), and CMPD9 (**15**, Figure 5) which possess –COOH, –(CH2)4–CH3, and –CH3 substitutions, respectively, on the 2′ position of the 2,4-dihydroxyphenyl ring of deoxybenzoin. Results showed that all three compounds increased the proliferation of the MCF-7 cell line and showed similar affinity for both ERα and ERβ receptors.

### 4.4. Hydrazide Derivatives

**Figure 6:**
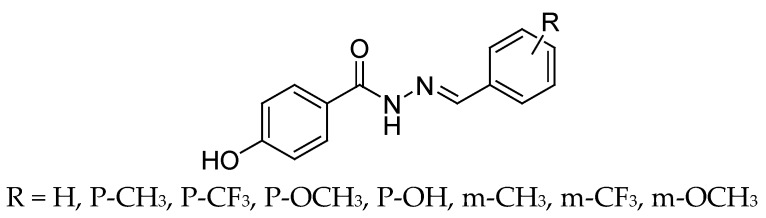
Hydrazide derivatives.

Recently hydrazide derivatives were patented as estrogen-related receptor (ERR) ligands. Figure 6 shows the structure of this series. Most of the compounds of this group were selective estrogen-related receptor modulators or co-activator selective ligands [41].

### 4.5. Fluoren-3-one Derivatives

**Figure 7:**
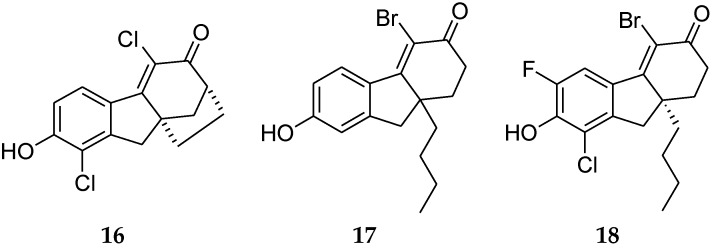
Fluoren-3-one analogues as selective ERβ.

Recently a new group of 7-hydroxyfluoren-3-one derivatives were reported as selective ERβ, such as compounds **16**–**18** (Figure 7) [42,43].

### 4.6. Triphenylethylene Coumarin

**Figure 8:**
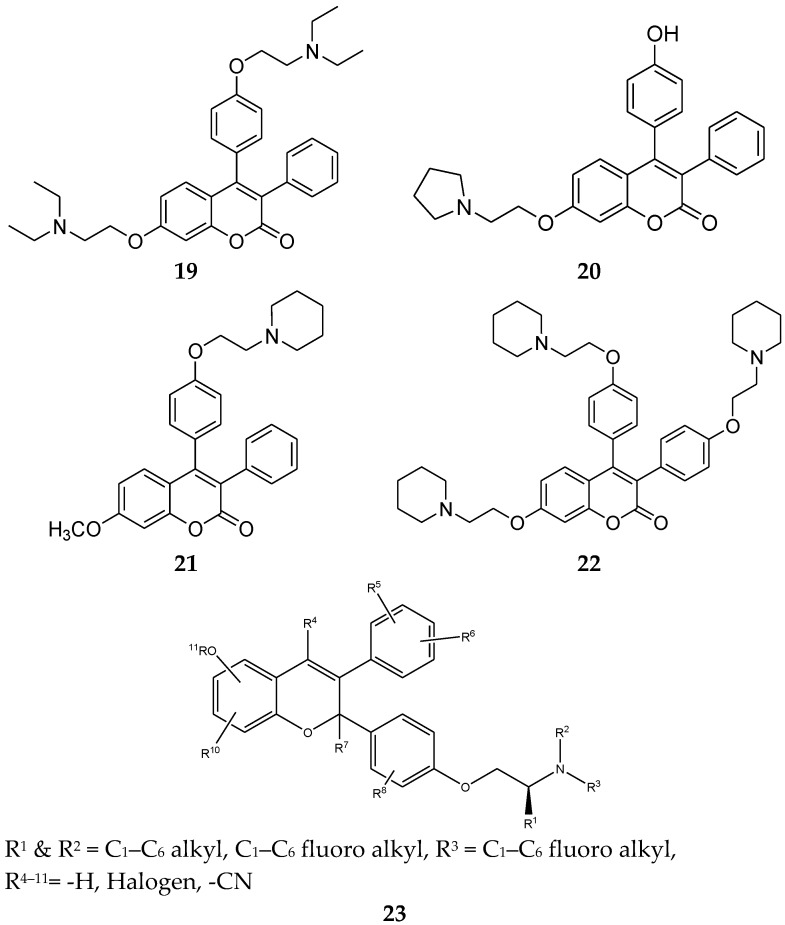
Triphenylethylene-coumarin hybrids.

Novel triphenylethylene–coumarin hybrid derivatives containing different types and increasing numbers of amino ethoxy side chains were reported and evaluated in 2013 [44]. These derivatives were subjected to anti-proliferative tests against five tumor cells and tamoxifen were used as a positive control. The derivatives displayed a broad-spectrum and good anti-proliferative activity. Between these compounds (**19**–**22**, Figure 8), compound **19** showed the best results. Therefore, these observations suggested that: (1) the number of the amino alkyl chain on 3,4-diphenylcoumarin had considerable impact on anti-proliferative activity, and two side chains were more preferable for this activity; (2) the weaker basic amino group on the side chain indicated a detrimental influence on anti-proliferative activity.

Also, in 2015, Smith et al. [45] patented some compounds that have a coumarin scaffold as estrogen receptor modulators. The general structure of **23** showed estrogen receptor antagonist activity and can also be an estrogen receptor degrader.

### 4.7. Combination of Anti-Estrogen and Receptor Tyrosine Kinase (RTK) Inhibitors

**Figure 9:**
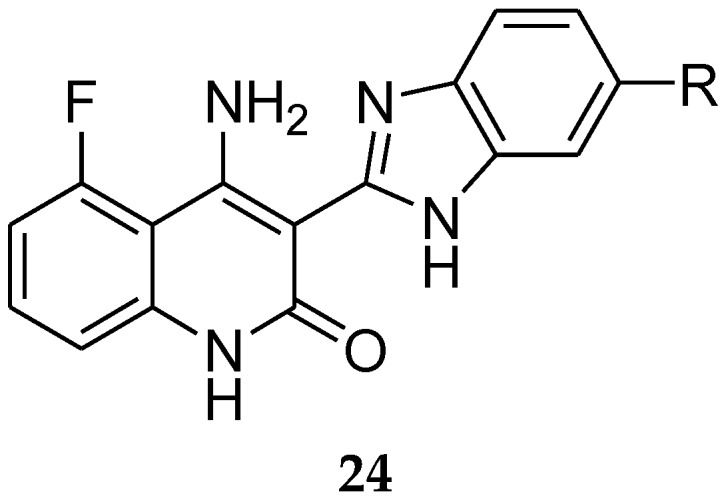
Receptor tyrosine kinase (RTK) inhibitors.

A combination of anti-estrogen and receptor tyrosine kinase (RTK) inhibitors was patented as a useful agent for the treatment of cancer by Novartis. Receptor tyrosine kinases (RTKs) are transmembrane polypeptides that regulate developmental cell growth. A combination of fulvestrant with RTK inhibitors such as compound **24** (Figure 9) displayed significant improvement in the treatment of proliferative diseases [46].

### 4.8. 1,1,2-Triarylolefine Derivatives

**Figure 10:**
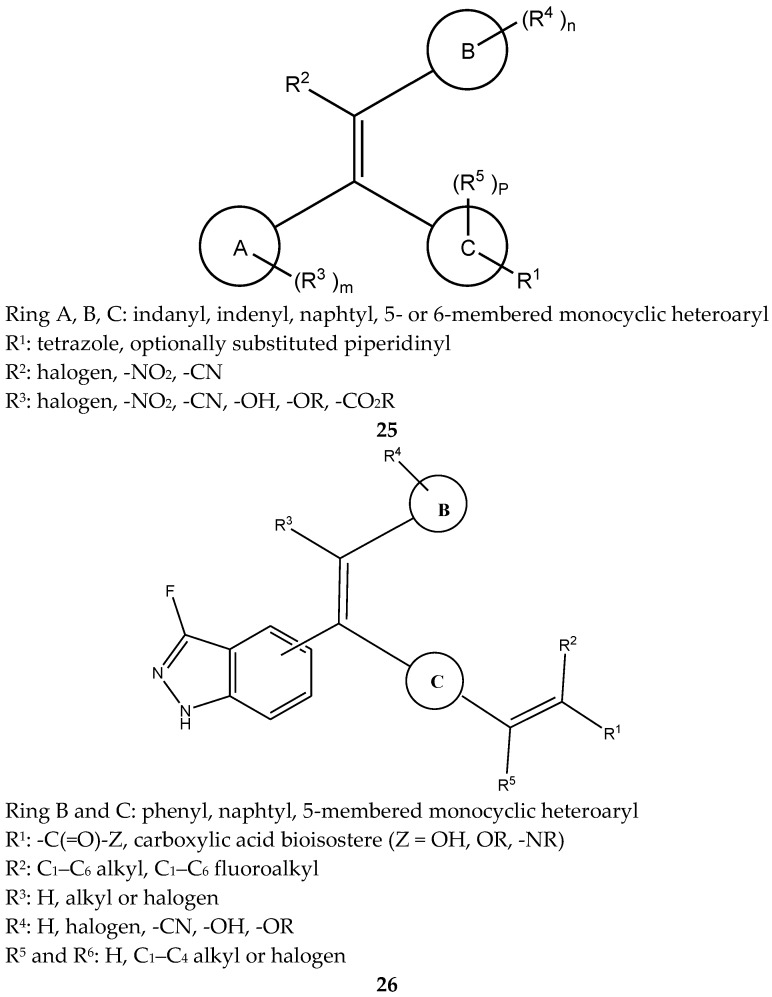
1,1,2-Triarylolefine.

A group of 1,1,2-triarylolefines were patented as estrogen receptor antagonists (**25**, Figure 10). Most of the synthesized compounds of this group had high anti-estrogenic effects with minimal or no estrogen receptor agonist activity [47].

Relating to the patent mentioned above, some new compounds were patented by Smith et al. as estrogen receptor modulators in 2015 [48,49]. This patent with formula **26** (Figure 10) showed that these compounds diminished the effects of estrogen at estrogen receptors and are useful for the treatment ER-related diseases. These compounds were tested in different cells related to estrogen and were tested in a clinical trial, demonstrating good results and high affinity to the estrogen receptor.

### 4.9. Aptamer Modulators of Estrogen Receptors

Shi Hua et al. [50] patented a nucleic acid aptamer molecule that includes a domain that binds to an estrogen receptor. Examples of aptamer molecules include those that bind to ERα and those that bind to ERβ. These compounds have a high affinity to the estrogen receptor; therefore, this strategy can be used for patients that have estrogen-dependent cancer.

## 5. Tamoxifen Analogues

**Figure 11:**
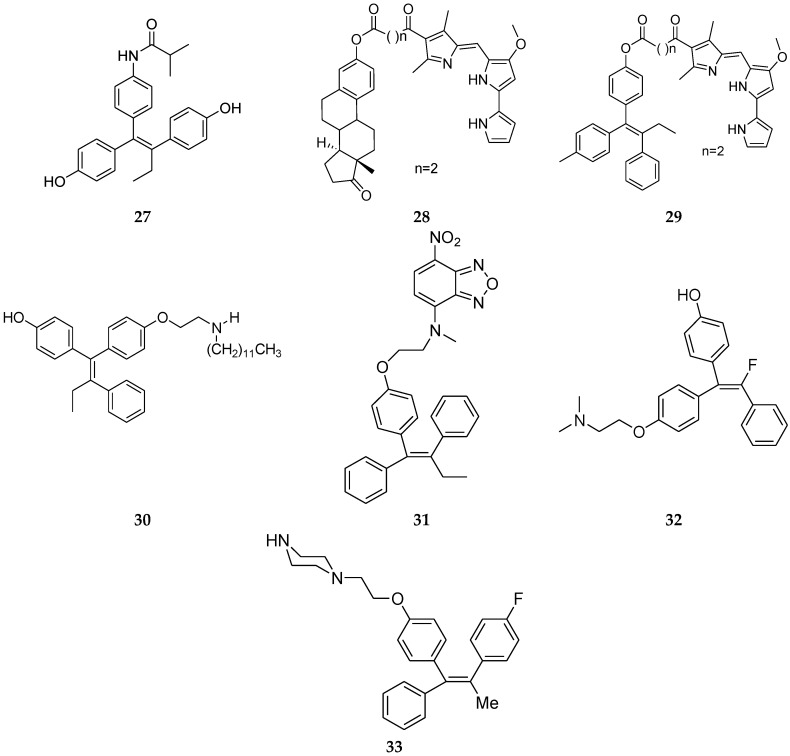
Tamoxifen analogues.

A new series of novel tamoxifene analogues were evaluated for their anti-proliferative activity on breast cancer (MCF-7) and their activity was comparable or even higher than tamoxifen. These derivatives maintained the triarylethylene skeleton of tamoxifen having OH groups at the para position of the phenyl rings of tamoxifen and substituted the side chain of tamoxifen with an amide side chain. One of the most active compounds in this series, such as **27** (Figure 11), had anti-proliferative activity about four times higher than tamoxifen [51].

**Figure 12:**
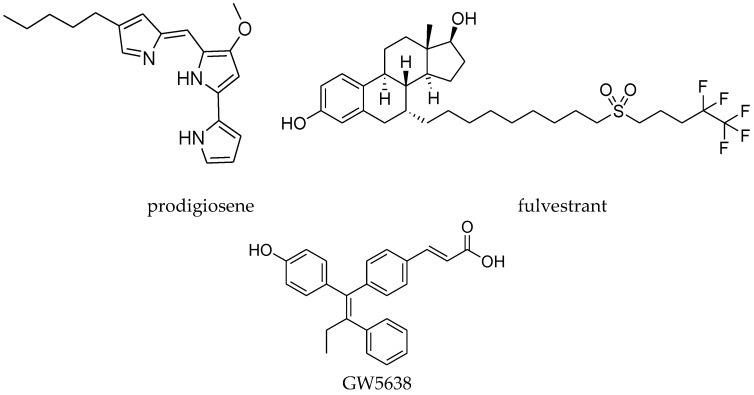
Estrogen ligands.

Recently, prodigiosene (Figure 12) conjugates of the tamoxifen analogue (**29,** Figure 11) and estrone (**28**, Figure 11) were reported. Prodigiosenes constitute a class of tripyrrolic compounds based on the natural product prodigiosin that share a common 4-methoxypyrrolyldipyrrin core unit. They have demonstrated anticancer activities [52,53]. Conjugating prodigiosenes to a molecule that already possesses selectivity toward the tumor should help to deliver the drug at a specific site [54,55,56,57,58,59,60,61,62,63]. Between these two compounds, the prodigiosin conjugates with the tamoxifen analogue (**29**, Figure 11) displayed excellent growth inhibition against the ER-positive cell line MCF-7, with better activity than tamoxifen itself against this cell line [64].

Another class of antagonists is the selective estrogen receptor down-regulators (SERDs). This class of compounds binds to ER and induces the rapid down-regulation of ER [65,66,67,68] and they have no agonistic activity in any tissues. SERDs are divided into two groups based on their chemical structures. One is steroidal compounds, such as fulvestrant (Figure 12), which has a steroidal structure with a long alkyl side chain at the 7α position of the 17ß-estradiol core [68,69,70]. The other group contains non-steroidal compounds, such as GW5638 (Figure 12). The structure of GW5638 is similar to tamoxifen and contains an acrylic acid side chain extending from the triphenylethylene core [71]. Shoda et al. [72] reported the design and synthesis of tamoxifen derivatives that induced down-regulation of the ER. Accordingly, a new series of tamoxifen derivatives with long alkyl chains was designed. Among them, compound **30** (Figure 11) had a strong antagonist effect.

In this regard, a series of novel fluorescent tamoxifen derivatives (FLTX1) were also reported as unique and SERM-like. Compound **31** (Figure 11), as a novel fluorescent (FLTX1), specifically labels intracellular tamoxifen-binding sites, including ERα, and displays unique pharmacological properties both in vitro and in vivo. FLTX1 exhibits the potent anti-estrogenic properties of tamoxifen in breast cancer cells without the estrogenic agonistic effect on the uterus [73]. 

Malo-Forest et al. [74] reported fluorinated derivatives of tamoxifen. The activities of these fluorinated analogues are similar or better than tamoxifen and among them, compound **32** (Figure 11) was the most active on the MCF7 cell line. In particular, as opposed to tamoxifen, both geometrical isomers behave similarly. This behavior may be due to in vitro isomerization of the compounds. 

Abdellatif et al. [75] also showed that replacement of the dimethylamino group in tamoxifen for piperazino or *N*-methylpiperazino and by changing the ethyl group by methyl substitution of the phenyl ring with a fluorine atom had excellent anti-proliferative activity on the MCF-7 cell line. Among these derivatives, compound **33** (Figure 11) showed the best results.

**Scheme 1:**
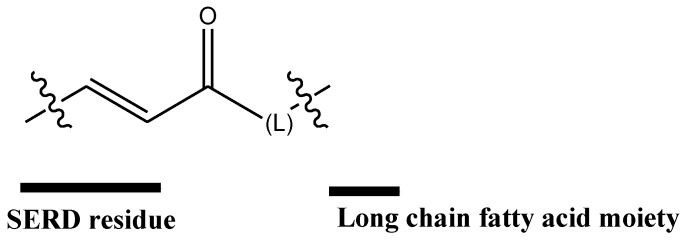
Covalently bonding a polycarboxylic fatty acid and SERD.

Cho et al. [76] patented compositions and methods for covalently bonding a polycarboxylic fatty acid and SERD as shown in Scheme 1. The conjugated compounds are useful for increasing solubility of the SERD as well as targeting the SERD to a solid tumor. Compounds have been shown to be interestingly efficacious against tamoxifen-resistant tumors. Currently, there is only one approved drug that binds to estrogen receptors for use in patients suffering from tamoxifen-resistant cancer.

**Figure 13:**
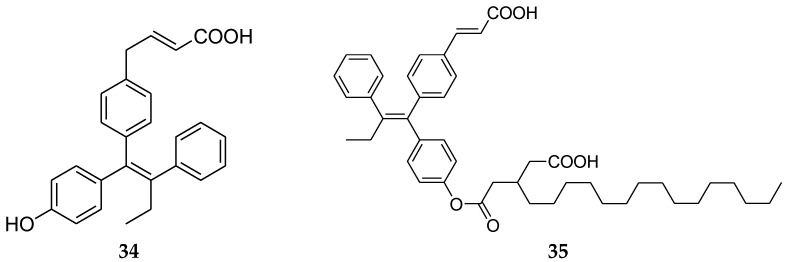
SERD (**34**) and fatty acid-SERD conjugate (**35**).

Preferably, the SERD is selected from the group consisting of compound **34** (Figure 13). In this group, results showed that compound **35** had the best activity, being even more potent than fulvestrant.

## 6. Carborane Analogues

**Figure 14:**
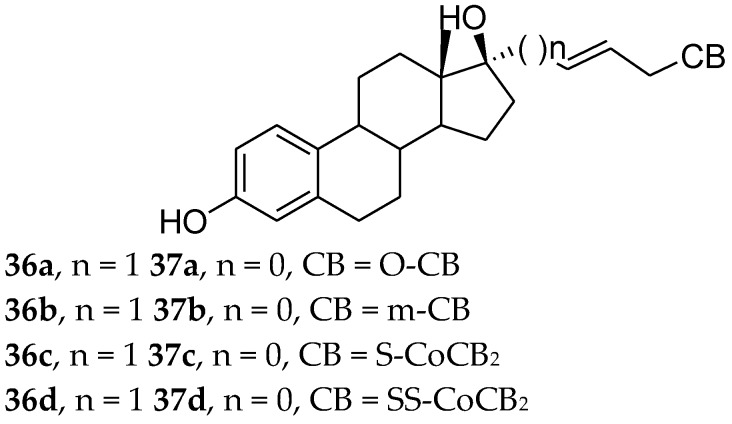

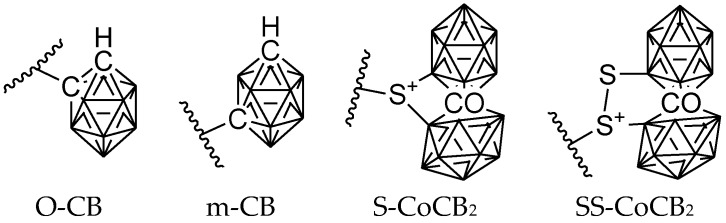
17α-Carboranylalkylestradiols.

Carboranes are man-made compounds that have no counterparts in nature. Their unique bonding, structure, and properties predetermine them to be used and tested in many applications. During the last decade it has been shown that 17α-substituted arylestradiol sharing a lipophilic aromatic moiety displayed high affinity for binding to ERα and ERβ receptors [61,77,78,79,80,81,82,83]. As a result, Sedlak et al. [84] reported a new series of 17α-(carboranylalkyl)estradiols as ligands for estrogen receptors α and β. Results showed that all of the tested compounds (Figure 14) had estrogenic activity, although none of them were able to fully activate either ER like 17β-estradiol. Relative transcription activity (RTA) is shown in Table 1. Results also showed that compound **37a** is 30 times more selective for ERα than ERβ and it is the most potent ligand for ERα among the tested compounds. They also showed that the length and the position of the double bond in the alkenyl linker are important for the affinity of the ligand to the receptor. Smaller carborane groups require shorter linkers to efficiently activate ERs.

**Figure 15:**
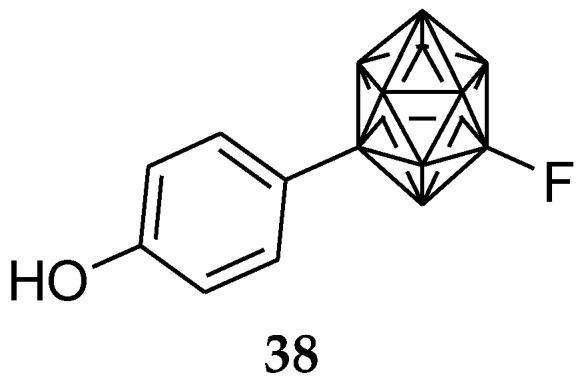
Fluorinated carborane as ERβ selective.

A new series of fluorinated carboranyl phenols were also reported as ERβ selective ligands. Compound **38** (Figure 15) in this series displayed 8.5-fold greater selectivity for ERβ. Therefore, introduction of a fluorine atom at the 9-position of the m-carborane cage reduced the ERα binding affinity [85].

**Figure 16:**
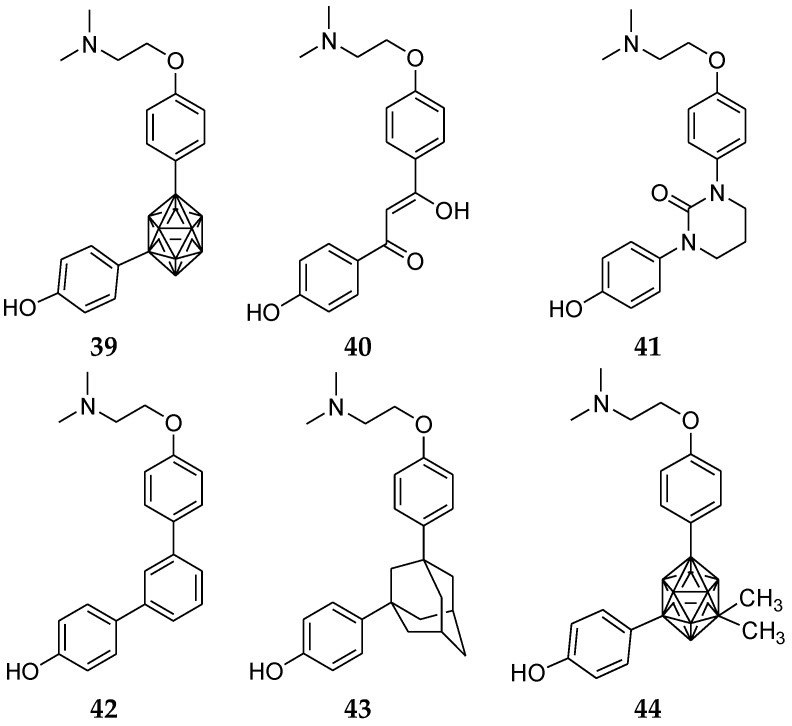
Hydrophobic bent-core structures.

Ogawa et al. [86] designed and synthesized novel estrogen receptor modulators containing various hydrophobic bent-core structures. Previously discovered m-carborane derivative (**39**, Figure 16) displayed ER partial agonistic activity in ERα trans activation assays. In this series, compounds with pseudo cyclic, tetrahydropyrimidinone, m-benzene, adamantine, and 9,10-dimethyl-m-carborane were synthesized. Among them, compound **44** with a 9,10-dimethyl-m-carborane cage showed a greater ER-binding affinity than compound **39** with two methyl groups into the m-carborane cage. Compound **39** had an increase of Hansch hydrophobic value (π value) [87,88].

## 7. Metal Complexes

**Figure 17:**
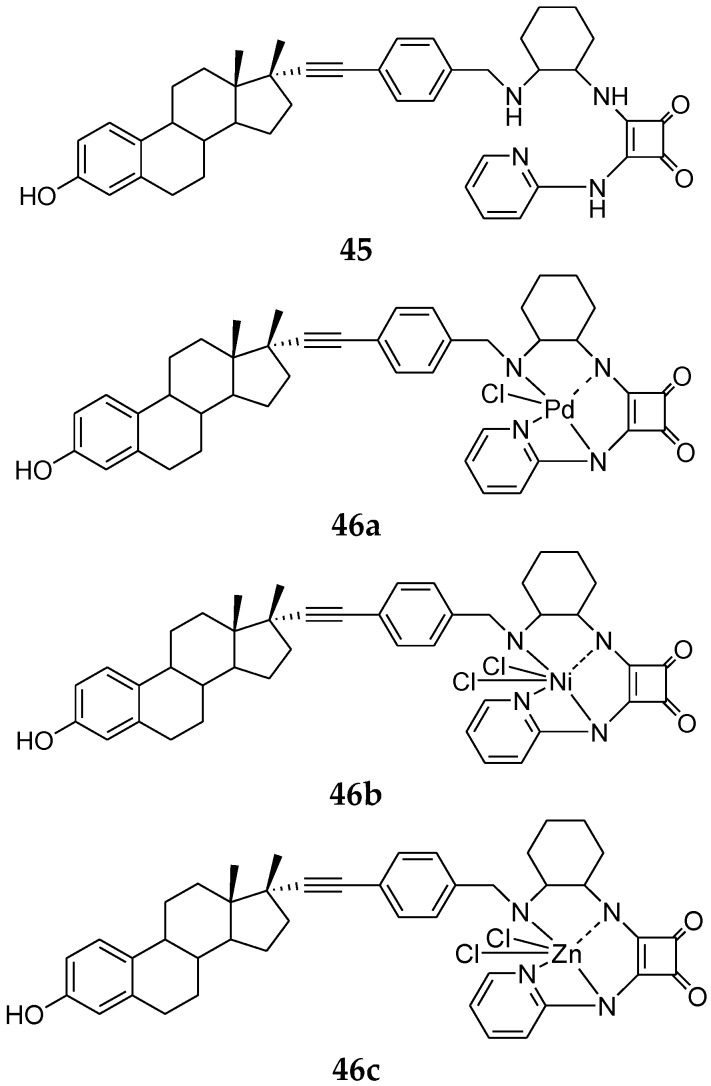
Novel type of targeting agents for ER.

Estrogenic steroid conjugates possessing metal chelates at the 17α-position represent an attractive delivery vector as a targeting strategy [89,90,91,92]. In this regard, Zhang et al. synthesized a new series of estrogen-derived metal complexes and applied the squaramide structure as the core of the metal binding unit. Results showed that all of the compounds in this series were agonists on ERα. Compared to the neutral free ligand (**45**, Figure 17), the binding affinity of compound **46a**–**c** for ERα increased, while the binding affinity of **46a**–**c** for ERβ all decreased [93].

**Figure 18:**
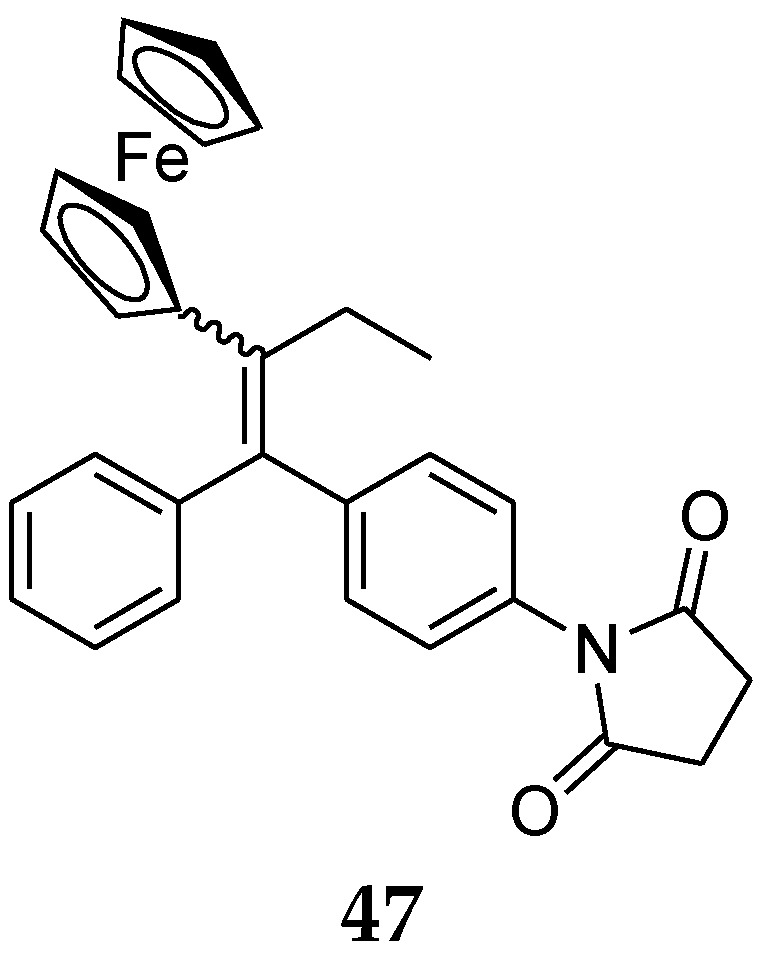
Ferrocenyl compound.

Recently, new ferrocenyl compounds with different alkyl chain lengths were synthesized and evaluated against hormone-dependent breast cancer cells. Seven new ferrocenyl compounds having different chain lengths (suberic, adipic, succinic) were synthesized and all of them displayed strong anti-proliferative activity against hormone-dependent and independent breast cancer cell lines. Interestingly, among this series, compound **47**, having a succinimide group (Figure 18), was the most active compound against hormone-dependent MCF-7 breast cancer cells, presumably owing to an antagonist effect on ERα [94].

**Figure 19:**
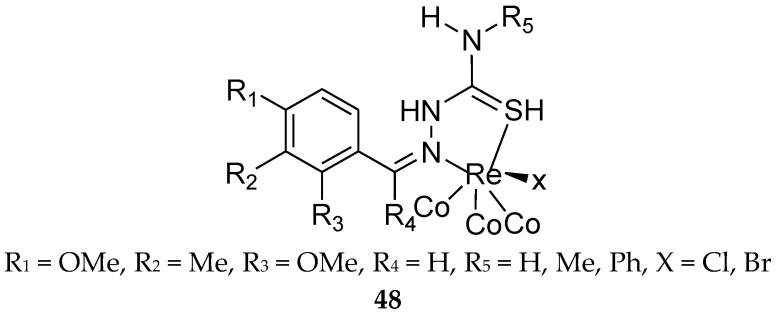
Rhenium(Ι) thiosemicarbazone complexes.

In 2014, some thiosemicarbazone ligands and their rhenium(I) carbonyl complexes were synthesized, characterized, and their estrogen receptor binding affinities were determined (**48**, Figure 19). In general, all compounds displayed values of less than 50% for binding affinity for both estrogen receptor subtypes.

The results indicated that the type of halogen on the rhenium atom does not seem to have an effect on the affinity of the complex for the receptor, but the hydroxyl group at the para position of phenyl ring plays a positive role. In any case, the nature of the R_3_ and R_5_ groups seems to be more significant [95].

## 8. Conclusions

Since activation of ERα is associated with proliferative responses in the mammary gland and in the uterus, the development of selective ERα antagonists or selective ERα ligands with mixed tissue-selective agonist/antagonist activity is a promising strategy for the production of new therapeutic agents. In contrast, the therapeutic potentials of ERβ agonists for the treatment of other pathologies such as prostate cancer or cardiovascular diseases attract researchers to develop selective ERβ agonists as safe and efficacious drugs. This review provides an overview and update of compounds that have been recently reported as estrogen receptor ligands, with a particular focus on their structure-activity relationships and their potential clinical applications. The compounds were classified based on chemical structure in steroidal and non-steroidal ligands. Atypical ER ligands including carborane analogues and estrogenic metal complexes were also described.

SERMs are compounds that exhibit tissue-specific estrogen receptor agonist or antagonist activity [2]. They represent a diverse group of molecules with varying levels of estrogen agonist and antagonist activity in different tissues. With unique and different patterns of ER subtype expression seen in the breast, bone, CNS, and cardiovascular system, ER ligands including SERMs can exert a wide range of physiological effects related to both pathological and therapeutic processes. The most important clinical successes achieved in the ER ligand field over the past decades mainly involve SERMs or ERα antagonists which are used in the treatment of estrogen-related cancers such as breast cancer, osteoporosis, and cardiovascular diseases [47]. The classical treatment in ER-positive breast cancer is oral tamoxifen as a first-generation SERM. However, over time or by chance mutation, ER-positive breast cancer not only becomes resistant to tamoxifen, as tamoxifen becomes an agonist which induces proliferation. Unlike SERMs like tamoxifen, SERDs are not ER agonists which limit side effects throughout the body such as endometrial hyperplasia or uterotrophy and bone demineralization which make them much more tolerable therapies. Unfortunately, there is only one approved hormonal estrogen receptor-targeted medication for tamoxifen-resistant cancer, the SERD fulvestrant. Due to solubility issues of fulvestrant, it has to be prepared in oil and used as an IM injection which is extremely painful and unpleasant for patients. Therefore, there is a need to develop new SERDs with oral activity as alternative treatment for patients suffering from tamoxifen-resistant cancers. Conjugation of a SERD and a polycarboxylic fatty acid is a good strategy to form fatty acid-drug conjugate compounds. The conjugate compounds are useful for increasing solubility of the SERDs as well as targeting the SERD to solid tumors. In particular, the compounds have been shown to be interestingly efficacious against tamoxifen-resistant tumors. The authors believe that in the near future, several new SERDs will be introduced in this field for the treatment of tamoxifen-resistant cancers. In fact, new orally bioavailable SERDs hold promise as a next generation therapy for the treatment of ER-positive breast cancer as monotherapy as well as in combination with agents that target other pathways involved in endocrine-resistant states. In addition to SERDs, a great deal of attention is presently being paid to ERβ agonists. Recently, a remarkable rise of interest in developing ER modulators displaying the same beneficial effects of estrogens without the adverse side effects was observed after the discovery of a second ER subtype, ERβ. Therefore, the recent literature has focused on selective ERβ ligands as highly promising targets for the treatment of some types of cancer, as well as for cardiovascular and inflammatory bowel diseases. Because of the high therapeutic potential of ERβ ligands, we should expect that in the coming years more ERβ-selective agonists will be launched in the market for the treatment of different pathologies such as breast cancer, prostate cancer, colorectal cancer, cardiovascular diseases, and neurodegenerative diseases.

## Figures and Tables

**Table 1:** Activation of steroid receptors by carboranyl compounds.

Compound	RTA(%) ERα	RTA(%) ERβ
**Agonist**	100	100
**36a**	24	26
**36b**	52	35
**36c**	20	16
**36d**	53	43
**37a**	58	40
**37b**	71	63
**37c**	16	14
**37d**	31	27
